# Categorical Data Analysis Using a Skewed Weibull Regression Model

**DOI:** 10.3390/e20030176

**Published:** 2018-03-07

**Authors:** Renault Caron, Debajyoti Sinha, Dipak K. Dey, Adriano Polpo

**Affiliations:** 1 Department of Statistics, Federal University of São Carlos, São Carlos 13565-905, Brazil; 2 Department of Statistics, Florida State University, Tallahassee, FL 32306, USA; 3 Department of Statistics, University of Connecticut, Storrs, CT 06269, USA

**Keywords:** asymmetric model, binomial response, multinomial response, skewed link, Weibull distribution

## Abstract

In this paper, we present a Weibull link (skewed) model for categorical response data arising from binomial as well as multinomial model. We show that, for such types of categorical data, the most commonly used models (logit, probit and complementary log–log) can be obtained as limiting cases. We further compare the proposed model with some other asymmetrical models. The Bayesian as well as frequentist estimation procedures for binomial and multinomial data responses are presented in detail. The analysis of two datasets to show the efficiency of the proposed model is performed.

## 1. Introduction

The statistical problem of estimating binary response variables is very important in many areas including social science, biology and economics [1]. The vast bibliography of categorical data presents the big evolution of the methods that handle appropriately binary and polychotomous data. More details can be found in Agresti [2]. Generalized linear model (GLM) has a wide range of tools in regression for count data [3]. Two important and commonly used symmetric link functions in GLM are the logit and probit links [4]. Many studies have investigated the limitations of these symmetric link functions. It is well accepted that when the probability of the binary response approaches 0 at a different rate from the rate (as a function of covariate) it approaches 1, symmetric link functions cannot be appropriate [5]. Many parameteric classes of link functions are in the literature, including the power transform of logit link by Aranda-Ordaz [6] and the a general link class of Chen et al. [5]. Other works with one-parameter class include Guerrero and Johnson [7], Morgan [8], Whittmore [9] and a host of others. Existing models for two-parameter families include Stukel [10], Prentice [11], Pregibon [12], Czado [13] and Czado [14].

Stukel’s model with transformation of both tails of logit link is very general and can approximate many important links including probit, logit and complementary log–log. However, the Bayesian analysis of Stukel’s model is not straightforward to implement, particularly in presence of multiple covariates and noninformative improper priors. The model proposed by Chen et al. [5], which includes the skew-probit model, uses a latent variable approach [15] that is convenient for sampling from the posterior distribution. Using the Albert and Chib [15] technique, Kim et al. [16] proposed the generalized t-link models, Naranjo et al. [17] proposed the asymmetric exponential power (AEP) model, and Rubio and Liseo [18] discuss the Jeffreys prior for skew-symmetric models. However the frequentist analysis for these models are not trivial. For the skew-probit model, The existence of the maximum likelihood estimator (MLE) of the linear regression parameters (β) can be proved only under the restrictive condition that the skewness parameter of the link function is known [19].

The majority of the works in literature are devoted to the models for binary response data. For the case of multinomial data, the multinomial extension of the logit link [20] (Chapter 8) and associated inference tools are simple to perform, and the marginal distribution of each component preserves the logit link. As mentioned before, the symmetric link may not be always appropriate. This is even clearer in multinomial data, where the sense of symmetric link is not simple to state. Generally, some categories has few observations when compared to the other ones, suggesting the idea of asymmetric distribution. We are also not aware of any model with asymmetric link function for multinomial data.

Caron and Polpo [21] briefly suggested an asymmetrical link function, called Weibull link, exclusively for binary response data. The use of Weibull distribution in survival/reliability analysis is well known. One important fact is the simplicity of the distribution, which has an analytic expression for the distribution function. Our proposed link model, based in the Weibull distribution, preserves this simplicity and it is a good option for the analysis of binary data.

In this paper, we take the Bayesian route and extend their work to multinomial data. Further we present for the first time the associated Bayesian inference tools and explore the properties of the proposed link function. We show that the benefits of this model are as follows: (1) flexibility of the Weibull distribution; (2) logit, probit and complementary log–log links as limiting cases; (3) case of implementation of both frequentist and Bayesian inferences; and (4) a general extension to handle multinomial response. The implementation of the associated Markov chain Monte Carlo (MCMC) algorithm to sample from posterior distribution is not complicated. In addition, we develop an Empirical Bayes tool [22,23] to obtain the prior when there is no relevant prior information available to the statistician.

We illustrate the use of Weibull link via analysis of two following data examples. (1) For the experiment to study the potencies of three poisons [24], the main binary response is whether the insect is alive after being treated with assigned dose level. For this example, we compare our Weibull link model with other asymmetric and symmetric link models. (2) The main response of the study by Grazeffe et al. [25] is the multiple levels of DNA damage in circulating hemocytes of each adult snail irradiated with an assigned dose. This study is used to illustrate the analysis of multinomial response data under Weibull link model, and comparing the results with those obtained by Grazeffe et al. [25] using logistic regression.

The article is organized as follows. In Section 2, we present the Weibull model, its novel properties and some approximations of the link function. In Section 3, we present the estimation procedures using MLE as well as the Bayesian estimation. In Section 3, we also present the estimation procedure for multinomial response. Section 4 is devoted to illustrating the Weibull link for analyzing two real datasets, and comparison with other existing models. Finally, Section 5 presents some future considerations and final comments.

## 2. Weibull Regression Model

### 2.1. Link Function

Let $X={\left(1,X_{1},\ldots ,X_{r}\right)}^{\prime }$ be the design matrix, where 1 is a vector with all values equal to 1, j=1,…,r. We denote the vector of binary response variable as Y. Similar to GLM, our interest lies in modeling the probability $\mathrm{Pr}\left[Y_{i}=1|\eta_{i}\right]=\mu \left(\eta_{i}\right)=E\left(Y_{i}\right)$ as $\mathrm{Pr}\left[Y_{i}=1|\eta_{i}\right]=g^{-1}\left(\eta_{i}\right)$, i=1,…,n, where η=βX, $\beta =(\beta_{0},\;\beta_{1},\;\ldots ,\beta_{r})$ are the linear coefficients, and g(·) is the link function. The link function relates the covariates X with the mean response μ=E(Y∣X). In this case, the $g^{-1}$ is a cumulative distribution function (cdf) on the real line. Our interest is a link function that can accommodate symmetric and asymmetric tails which has a simple parameteric functional form, and can be easily tractable. To obtain these goals, we use the cdf of Weibull distribution

(1) $$F\left(\eta \right)=1-\exp\left\{-{\left(\eta -\alpha \right)}^{\gamma }\right\}I_{\left(\eta >\alpha \right)},$$

for $g^{-1}$, where α∈R is the location/threshold parameter, γ>0 is the shape parameter, and $I_{\left(\eta >\alpha \right)}$ is the indicator of η>α. $I_{A}$ is the indicator function of event *A*, that is $I_{A}=1$ and $I_{A^{c}}=0$.

Alternatively, the Weibull link function is defined as

(2) $$\begin{array}{ccc} \eta = & g(\mu ) & ={\left[-\log\left(1-\mu \right)\right]}^{\frac{1}{\gamma }},\\ \mu = & g^{-1}\left(\eta \right) & =1-\exp\{-\eta^{\gamma }\}, \end{array}$$

where μ(η)=E(Y∣η)≥0, γ>0, and η>0.

**Figure 1:**
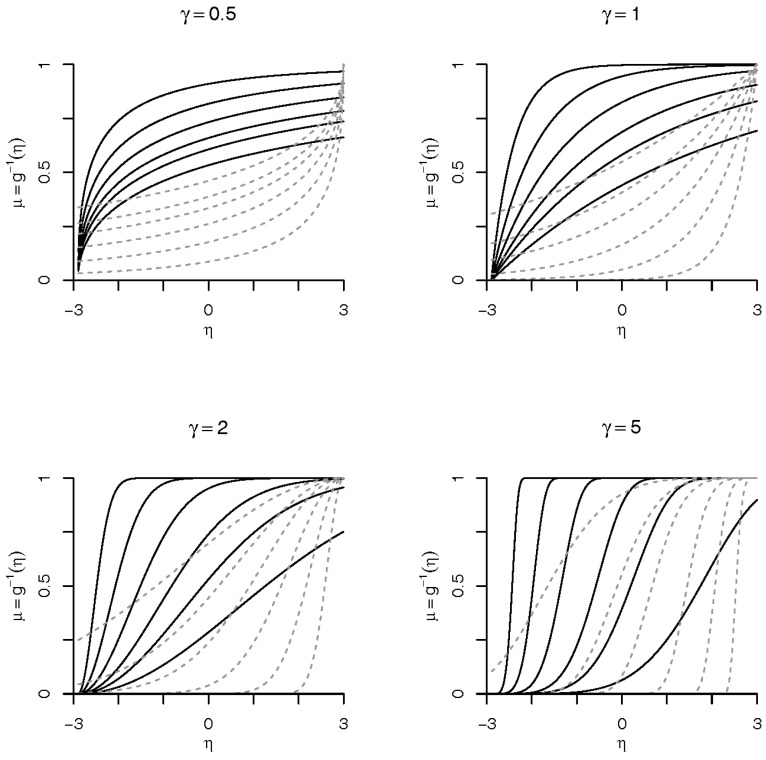
Forms of Weibull link. Solid lines are for the Weibull link and dashed lines are for the reflected Weibull link. We have used $\eta =\beta_{0}+\beta_{1}x$, where *x* is a grid in (0.0001,5.9), $\beta_{0}=-2.9$, and for $\beta_{1}$ we have considered the values 0.2, 0.3, 0.4, 0.6, and 2.

Note that, in the above parameterization, the restriction of η>0 is not a problem because the parameter $\beta_{0}$ plays the role of both the location/threshold parameter α and the intercept of linear predictor η=βX. By doing this, we avoid the identifiability problem in estimation of $\beta_{0}$, also we have a more parsimonious model. The skewness of the Weibull link depends only on the parameter γ, and can be evaluated by $\left(\Gamma_{3}-3\Gamma_{2}\Gamma_{1}+2\Gamma_{1}^{3}\right)/{\left(\Gamma_{2}-\Gamma_{1}^{2}\right)}^{3/2}$, where $\Gamma_{j}=\Gamma \left(1+j/\gamma \right)$ and Γ(·) is the Gamma function. The skewness lies in the interval (−1.1395,∞). We also evaluated the Arnold–Groeneveld (AG) skewness measure [26], which is a skewness measure related to the mode of a distribution. Again, the AG skewness depends only on the parameter γ, and can be evaluated as 2exp{(1−γ)/γ}−1, and lies in the interval (−0.26424,∞). However, sometimes, a model with skewness lower than −1.1395 is desired; in this case, we can use the reflected Weibull distribution to define the link as $\mu =g^{-1}\left(\omega \right)=\exp\left\{-\eta^{\gamma }\right\}$, and the skewness lies in the interval (−∞,1.1395). The different forms of Weibull link are shown in Figure 1 with solid line for the Weibull link and dashed line for the reflected Weibull link.

### 2.2. Special Cases

**Figure 2:**
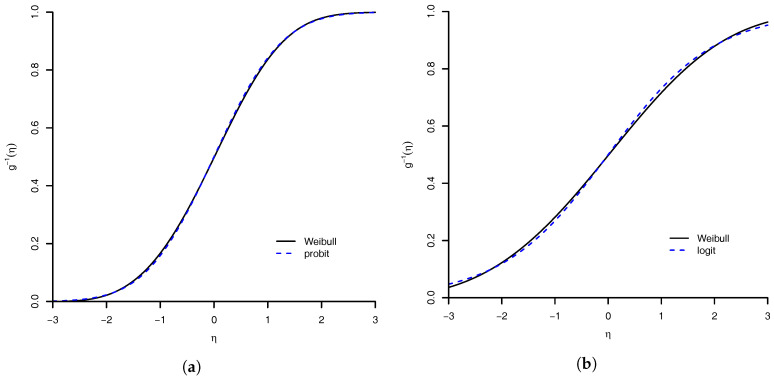
Similarity of Weibull link with: (**a**) probit link; and (**b**) logit link. The maximum absolute distance between Weibull link and probit link is 0.0078, and with logit link is 0.0148.

The choice of the Weibull distribution as link function is due to its flexible properties. Rinne [27] (Chapter 3) discusses the various properties of Weibull along with Weibull distribution as approximation to some symmetrical distributions. We highlight the relations of Weibull with the normal and logistic distributions, because they explain the relations of Weibull link with probit and logit link functions. Based on results of Rinne [27], we have

$$\begin{array}{ccc} g_{1}^{-1}\left(\eta \right) & = & 1-\exp\left\{-{\left(0.90114+0.27787\eta \right)}^{3.60235}\right\}\approx \Phi \left(\eta \right),\\ g_{2}^{-1}\left(\eta \right) & = & 1-\exp\left\{-{\left(0.89864+0.16957\eta \right)}^{3.50215}\right\}\approx \frac{\exp(\eta )}{1+\exp(\eta )}, \end{array}$$

where Φ is the distribution function of the standard normal distribution. These results show that Weibull link can approximate the probit link and the logit link. The degrees of these approximations are illustrated in Figure 2.

We have the following proposition for another important case of link, the complementary log–log link [4].

**Proposition** **1.** *The complementary log–log link defined by $g^{-1}\left(\eta \right)=1-\exp\left\{-\exp\left(\eta \right)\right\}$ is a limiting case of the Weibull link because*

(3) $$\lim_{\gamma \to \infty }\left\{1-\exp\left[-{\left(1+\frac{\eta }{\gamma }\right)}^{\gamma }\right]\right\}=1-\exp\left\{-\exp\left(\eta \right)\right\}.$$

**Proof.**  Taking α=−1 in Equation (1) and dividing η by γ, without loss of generality, we can rewrite the Weibull link given in Equation (2) as:

$$g^{-1}\left(\eta \right)=1-\exp\left\{-{\left(1+\frac{\eta }{\gamma }\right)}^{\gamma }\right\}.$$

Now, taking the limit γ→∞ of $g^{-1}\left(\eta \right)$ completes the proof. □

**Figure 3:**
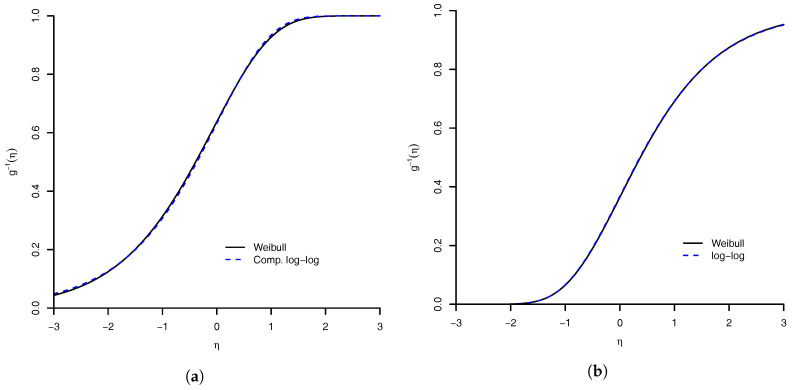
Similarity of Weibull link with: (**a**) complementary log–log link; and (**b**) log–log link. The maximum absolute distance between Weibull link and complementary log–log link is 0.0082 (the value of γ parameter was 21.03), and with log–log link is 0.0031 (the value of γ parameter was 114.45).

Given this result, we can say that for a dataset when the estimated value of γ is large then the complementary log–log link should be appropriate. Using the reflected Weibull link, we have a similar result with the log–log link, defined as $g^{-1}\left(\eta \right)=\exp\left\{-\exp\left(-\eta \right)\right\}$ [4]. The complementary log–log and log–log link as limiting cases are illustrated in Figure 3.

## 3. Estimation

### 3.1. Binomial Data

Consider a sample of size *n* from the binary variable/response *Y*, with $\mathrm{Pr}[Y_{i}=1]=$$p_{i}$ for i=1,…,n. We denote the observed data as D={n,Y=y,X=x}, where $y=(y_{1},\ldots ,y_{n})$ is the observed vector of $Y=(Y_{1},\ldots ,Y_{n})$, and $x={\left(1,x_{1},\ldots ,x_{r}\right)}^{\prime }$, is the observed covariate matrix of $X={\left(1,X_{1},\ldots ,X_{r}\right)}^{\prime }$. The likelihood function for the Weibull link can be written as

(4) $$\begin{array}{ccc} \;L\left(\beta ,\gamma |D\right) & \;\propto & \;\prod_{i=1}^{n}{p_{i}}^{y_{i}}{\left(1-p_{i}\right)}^{1-y_{i}}\\ & \;\propto & \;\prod_{i=1}^{n}{\left[1-\exp\left\{-\eta_{i}^{\gamma }\right\}\right]}^{y_{i}}\;{\left[\exp\left\{-\eta_{i}^{\gamma }\right\}\right]}^{1-y_{i}}\;, \end{array}$$

and the log-likelihood as

(5) $$l\left(\beta ,\gamma |D\right)\propto \sum_{i=1}^{n}\left[y_{i}\log\left\{1-\exp\left(-\eta_{i}^{\gamma }\right)\right\}-\left(1-y_{i}\right)\eta_{i}^{\gamma }\right],$$

where $\eta_{i}$ is the *i*-th element of the vector η=βX, and β, γ are the parameters to be estimated.

A numerical method such as Nelder and Mead [28] can be used to obtain the MLE for (β,γ). The expression of the gradient vector and Hessian matrix are given in Appendix A. Using the gradient vector and Hessian matrix, it is simple to implement a Newton–Raphson algorithm to obtain the MLE. As initial guesses for the numerical algorithm, we suggest to use the estimator ${\tilde{\beta }}_{i,probit}$ under probit model for $\beta_{i}$ (i≠0), ${\tilde{\beta }}_{0,guess}=-\min\left({\tilde{\beta }}_{probit}x\right)+0.001$ for $\beta_{0}$, and 3.60235 for γ. The initial guesses (β,γ) can be interpreted as the Weibull link being an approximate probit link.

For the Bayesian analysis, the posterior density is

(6) p(β,γ∣D)∝L(β,γ∣D)p(β,γ),

where p(β,γ) is the joint prior. We suggest using the hierarchical Bayes model. Assuming the parameters are a priori independent, the first level of hierarchy has γ following a gamma distribution with mean $m_{\gamma }$ and variance $v_{\gamma }$, and β with multivariate normal distribution with mean vector $m_{\beta }$ and covariance matrix $v_{\beta }I$, where I is the identity matrix. The values of $v_{\gamma }$ and $v_{\beta }$ are fixed, and for $m_{\gamma }$ and $m_{\beta }$ we consider a prior, that is $p\left(m_{\gamma }\right)=p\left(m_{\beta }\right)\propto 1$. Arguably, we can use the mode of the integrated likelihood of ($m_{\gamma }$, $m_{\beta }$) to determine a prior distribution [23]. The hyper-parameters $v_{\gamma }$ and $v_{\beta }$ are viewed as prior precision parameters. The EM (Expectation–Maximization) algorithm [29] can be used to obtain the estimates of $m_{\gamma }$ and $m_{\beta }$. The MCMC procedure is used to generate a sample from the posterior distribution. For the MCMC procedure, we used a Gibbs sampler with Metropolis–Hasting. The convergence of the chain was monitored using ergodic means. We omit the details about these computational tools because they are already well known tools and are not the main subject of the present paper. In addition, it was not necessary to develop any special scheme to sample from the posterior chain.

Another advantage of the Weibull link is that the posterior distributions are proper even when we use a wide range of non-informative priors. The Jeffreys’ prior for the parameter β has the form $p\left(\beta |\gamma \right)\propto {\left|I\left(\beta |\gamma \right)\right|}^{1/2}$, where the Fisher information matrix I(β∣γ) can be obtained by taking the expectation of the Hessian matrix given in Appendix A.

Considering the improper prior p(β)∝1, and the non-informative prior $p\left(\gamma \right)\propto 1/\gamma^{c}$, for γ>1 and c>1 a known constant [30], we have the non-informative prior distribution

(7) $$p\left(\beta ,\gamma \right)\propto p\left(\beta \right)p\left(\gamma \right)\propto \frac{1}{\gamma^{c}}.$$

With this constraint (in the parameter γ of the Weibull link), the skewness lies in the interval (−1.1395,2], which is still a flexible link. For the improper prior of Equation (7), the propriety of the resulting posterior distribution in Equation (6) is stated in Theorem 1.

**Theorem** **1.** Let $z_{i}=-1$ when $y_{i}=0$ and $z_{i}=1$ when $y_{i}=1$, and $X^{*}$ be the matrix with rows $z_{i}x_{i}^{\prime }$. Suppose that the design matrix X is of full rank, and there exists a positive vector $a={\left(a_{1},\ldots ,a_{n}\right)}^{\prime }\in R^{n}$, with $a_{i}>0$, for i=1,…,n, such that ${X^{*}}^{\prime }a=0$, under the non-informative prior of Equation (7), then the posterior density Equation (6) is proper.

**Proof.**  Let $u,u_{1},\ldots ,u_{n}$ be independent random variables with common Weibull distribution with shape parameter γ. For 0<k<∞, we have that $E(|u{|}^{k})=\Gamma \left(1+k/\gamma \right)<\infty$. Observing that 1−F(x)=E[I(u>x)] and F(x)=E[I(u≤x)], where I is an indicator function. Then, we have ${\left[F\left(x_{i}^{\prime }\beta \right)\right]}^{y_{i}}{\left[1-F\left(x_{i}^{\prime }\beta \right)\right]}^{1-y_{i}}\leq E\left(z_{i}u_{i}\geq z_{i}x_{i}^{\prime }\beta \right)$ and ${\left[F\left(x_{i}^{\prime }\beta \right)\right]}^{y_{i}}{\left[1-F\left(x_{i}^{\prime }\beta \right)\right]}^{1-y_{i}}\geq E\left(z_{i}u_{i}>z_{i}x_{i}^{\prime }\beta \right)$. Let $u^{*}=\left(z_{1}u_{1},\ldots ,z_{n}u_{n}\right)$. By the Fubini’s theorem, we get

$$\begin{array}{c} \int_{1}^{\infty }\int_{R^{k}}L\left(\beta ,\gamma |yX\right)\frac{1}{\gamma^{c}}d\beta d\gamma \\ \;=\int_{1}^{\infty }\frac{1}{\gamma^{c}}\int_{R^{n}}\int_{R^{k}}I\left(z_{i}u_{i}>z_{i}x_{i}^{\prime }\beta ,1\leq i\leq n\right)d\beta dF\left(u\right)d\gamma \\ \;=\int_{1}^{\infty }\frac{1}{\gamma^{c}}\int_{R^{n}}\int_{R^{k}}I\left(X^{*}\beta \leq u^{*}\right)d\beta dF\left(u\right)d\gamma . \end{array}$$

From Lemma 4.1 of Chen and Shao [31] there exists a constant *K* depending only on $X^{*}$ such that

$$\int_{R^{k}}I\left(X^{*}\beta \leq u^{*}\right)d\beta \leq K||u^{*}{\left|\right|}^{k},$$

which yields

$$\int_{1}^{\infty }\int_{R^{k}}L\left(\beta ,\gamma |yX\right)\frac{1}{\gamma^{c}}d\beta d\gamma <\infty ,$$

by $E(|u{|}^{k})<\infty$, and $\int_{1}^{\infty }1/\gamma^{c}d\gamma <\infty$ for c>1. □

This prior give a constraint in the parameter γ. However, any proper prior can be used with the proposed model, avoiding any constraint problem in the parameter γ.

### 3.2. Multinomial Data

For multinomial responses, we have that $Y_{i}\in \left\{1,\;\ldots ,\;K\right\}$, and $p_{k}=\mathrm{Pr}\left(Y_{i}=k\right)$, for k=1,…,K and $\sum_{j=1}^{K}p_{j}=1$. The logistic multinomial regression model consider a reference category, generally the category K, and have a link function

$$p_{k}=g_{k}^{-1}\left(\eta_{k}\right)=\frac{\exp(\eta_{k})}{1+\sum_{k=1}^{K-1}\exp\left(\eta_{k}\right)},\;k=1,\ldots ,K-1,\;\mathrm{and}\;p_{K}=1-\sum_{k=1}^{K-1}p_{k}=\frac{1}{1+\sum_{k=1}^{K-1}\exp\left(\eta_{k}\right)},$$

where $\eta_{k}=\beta_{k}X$, $\beta_{k}=\left\{\beta_{k0},\beta_{k1},\ldots ,\beta_{kr}\right\}$. The likelihood function for multinomial response data D is

(8) $$L\left(p|D\right)\propto \prod_{i=1}^{n}\prod_{k=1}^{K}p_{k}^{I(y_{i}=k)}=\prod_{k=1}^{K}p_{k}^{s_{k}},$$

where $s_{k}=\sum_{i=1}^{n}I\left(y_{i}=k\right)$, and $I_{A}$ is the indicator function of event *A*, that is $I_{A}=1$ and $I_{A^{c}}=0$. Note that $\sum_{k=1}^{K}s_{k}=n$.

Using a reparameterization [32] of p as $p_{1}=\theta_{1}$, $p_{k}=\theta_{k}\prod_{\ell =1}^{k-1}\left(1-\theta_{\ell }\right)$, for k=1,…,K−1, and $p_{K}=\prod_{\ell =1}^{K-1}\left(1-\theta_{\ell }\right)$ the likelihood function in Equation (8) can be rewritten as

(9) $$L\left(\theta |D\right)\propto \prod_{k=1}^{K-1}\theta_{k}^{s_{k}}{\left(1-\theta_{k}\right)}^{n-\sum_{\ell =1}^{k}s_{\ell }}=\prod_{k=1}^{K-1}L\left(\theta_{k}|D\right).$$

This shows that the estimation for multinomial data is equivalent to estimating K−1 binomial response models. We can consider any link function for binary data, taking $\theta_{k}=g^{-1}\left(\eta_{k}\right)$. For the MLE, we have ${\hat{p}}_{1}={\hat{\theta }}_{1}$, ${\hat{p}}_{k}={\hat{\theta }}_{k}\prod_{\ell =1}^{k-1}\left(1-{\hat{\theta }}_{\ell }\right)$, and ${\hat{p}}_{K}=\prod_{\ell =1}^{K-1}\left(1-{\hat{\theta }}_{\ell }\right)$. For Bayesian estimation, we generate a sample from the posterior distribution of each $\theta_{k}$, then we can do the transformation to obtain the estimators of p. Considering the Weibull link function, we need to generate a sample from the posterior of $\gamma_{k}$ and $\beta_{k}$, for each k=1,…,K−1, and then perform the proper transformation to obtain the sample from the posterior of $\theta_{k}$. In this case, the prior of $\theta_{k}$ can be viewed as a transformation of the priors of $\gamma_{k}$ and $\beta_{k}$. Thus, for both MLE and Bayesian estimator, we can use the procedures described in Section 3.1. The partition scheme presented to solve the multinomial model estimation is intuitive. For more details about the reparameterization used here, see Pereira and Stern [32].

### 3.3. Model Selection and Diagnostics

In the case of binomial data, to compare models within frequentist set up, we use the Akaike Information Criterion (AIC) [33] and the Bayesian Information Criterion (BIC) [34]. For Bayesian analysis, we use long established tool of Deviance Information Criterion (DIC) [35]. We omit the details of these popular tools for the sake of brevity. In addition, for Bayesian analysis we use the Pr(D|M) [36], where D is the observed data and *M* is the used model. Pr(D|M) is approximated by ${\left\{1/m\sum_{i=1}^{n}\mathrm{Pr}{\left(D|M,\theta_{i}\right)}^{-1}\right\}}^{-1}$, where $\theta_{i}$ is the *i*-th sample from the posterior distribution of θ under model *M*, given the data D. This measure is directly related to the Bayes Factor (BF). If the interest is to evaluate the $BF_{10}$ of the model $M_{1}$ against $M_{0}$, considering $\mathrm{Pr}\left(M_{0}\right)=\mathrm{Pr}\left(M_{1}\right)=0.5$, we have that $BF_{10}=\mathrm{Pr}\left(D|M_{1}\right)/\mathrm{Pr}\left(D|M_{0}\right)$. For both Bayesian and frequentist paradigms, we use: a version of Kolmogorov–Smirnov statistics (KS) as measure of goodness of fit (KS is defined as $KS=\max_{i}\left|y_{i}-\hat{y_{i}}\right|$, the maximum absolute error of the predicted and the observed frequencies, where $\hat{y_{i}}$ is the predicted value of $y_{i}$); the Mean Absolute Error (MAE) defined as $MAE=\frac{1}{n}\sum_{i=1}^{n}\left|y_{i}-\hat{y_{i}}\right|$; and the Brier Score (B-S) defined by Brier [37].

## 4. Data Example

In the data examples, we compare the proposed link function with some others links. Table 1 presents the link functions considered.

### 4.1. Binary Data Example

We analyze the study of relative potency of three different poisons: Rotenone, Deguelin and Mixture [24]. The experiment was to test the different poisons with different doses, with objective to understand the potency of the poisons. Five doses for rotenone, six doses for deguelin and six doses for the mixture were considered. For each dose and poison, around 50 insects were considered by observing how many insects were killed. The data are presented in Table 2. We consider that the response variable is binary with Y=1 representing the insect killed, and as covariates: $X_{1}$ as the log (Dose), $X_{2}$ as an indicator of Rotonone, and $X_{3}$ as an indicator of Deguelin. The mixture of poisons is considered as the reference poison (that is, $X_{2}=0$ and $X_{3}=0$). Our main objective is to find the model that better represents (fits) these Data. We are not looking for the “best” poison or dose.

**Figure 4:**
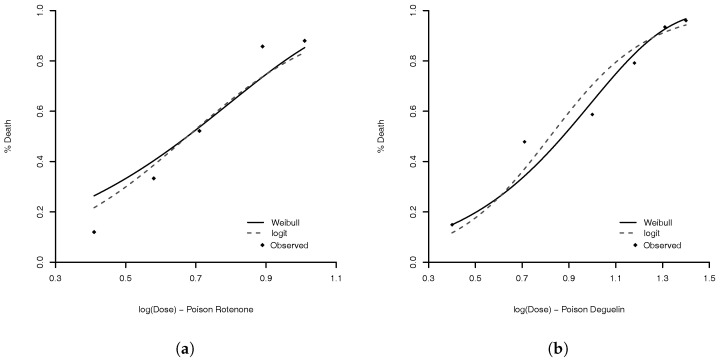

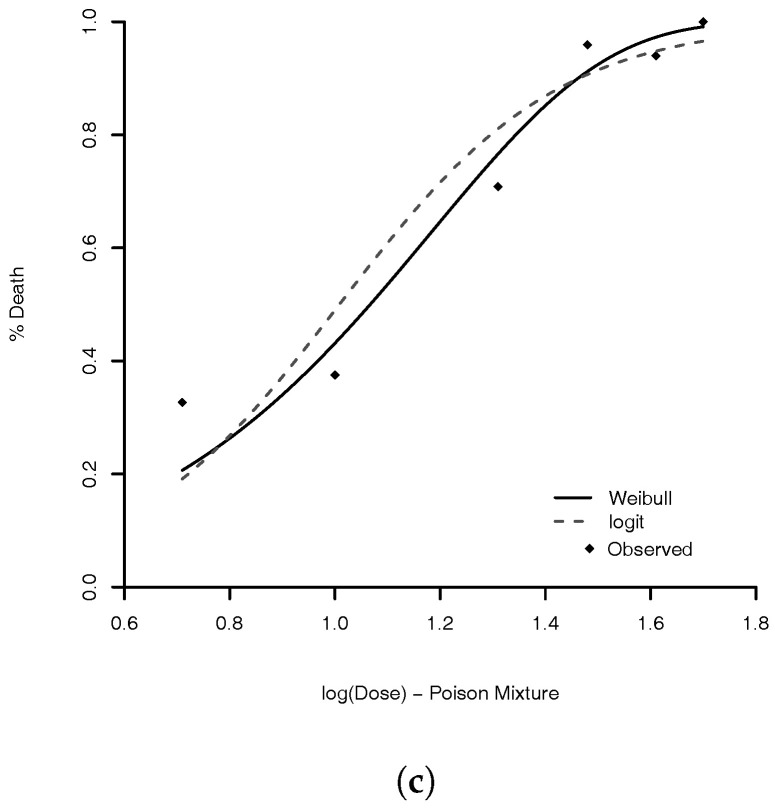
Comparison of MLE for Weibull (solid line) and logit link (dashed line) for three types of poisons: (**a**) Rotonone; (**b**) Deguelin; and (**c**) Mixture. The dots are the observed proportions.

We obtain the MLE for Weibull parameters and for comparison we also estimated the parameters of complementary log–log, Stukel, probit, logit, Aranda–Ordaz, and Prentice models. Table 3, presents some statistics of each model to compare them. The best models, based on AIC, are complementary log–log and Weibull. The advantage of the complementary log–log is that this model has one fewer parameter. However, $\hat{\gamma }=114.5084$ (Table 4), indicating that the Weibull model is going to the complementary log–log model. As expected, the Weibull model performs similar to the complementary log–log (see Proposition 1). The model with lowest KS is the logit model. The estimated logit and Weibull links are illustrated in Figure 4. The model with lowest MAE is the Stukel model. The estimated parameter values of Weibull logit, and Stukel models are given in Table 4.

Another important models are the skew-probit proposed by Chen et al. [5] and AEP proposed by Naranjo et al. [17]. To compare with the Weibull model we perform a Bayesian analysis for these models. The priors for the parameters of the Weibull model are the same as that described in Section 3.1. We use the values $v_{\gamma }=100$ and $v_{\beta }=25$. The estimated values for the hyper-parameters of first hierarchical level are $\hat{m_{\gamma }}=9.1089$ and $\hat{m_{\beta }}=\left(0.1588,0.8879,0.1261,-0.1717\right)$. For the priors of skew-probit model, we used a uniform distribution over the interval (−1,1) for the asymmetry parameter and independent normal distribution with mean 0 and variance 25 for each $\beta_{j}$, j=0,…,3. For the priors of AEP model, we used independent priors: gamma distribution with mean 1 and variance 100 for the parameters $\theta_{1}$ and $\theta_{2}$, and normal distribution with mean 0 and variance 25 for each $\beta_{j}$, j=0,…,3. Table 5 presents the model selection criteria (DIC, KS, MAE, B-S and Pr(D|M)) for the three competing models. For criteria DIC, KS, MAE and B-S, a smaller value indicates a better agreement between the model and observed data. For criterion Pr(D|M), a higher value indicates a better agreement between the model and observed data. We note that the Weibull model has better KS; AEP has the better DIC and MAE; and skew-probit has better Pr(D|M). The B-S was very similar for all models. For the three models, the posterior mean of relevant parameters are given in Table 6.

### 4.2. Multinomial Data Example

Grazeffe et al. [25] reported a study of DNA mutation of the cells of adult snails, each irradiated with a single dose of gamma radiation. They recorded four categories of DNA mutation with Y=1,2,3 and 4 representing no mutation, low, intermediate, and high DNA mutation respectively. The snails are randomized into five different dose levels with X∈{0,2.5,5,10,20}. The data are presented in Table 1 of Grazeffe et al. [25]. The objective is to compare effects of different dose levels on DNA mutation *Y* (Y=1 for C0, Y=2 for C1, Y=3 for C2 and Y=4 for C3). We illustrate the use of Weibull link model, under frequentist approach, for analysis of this study with multinomial responses. Further, in Table 10, we compare our estimates of Pr[Y=k∣x] with those obtained by Grazeffe et al. [25] based on the logit link model, and the other models discussed here.

For a proper comparison with previous method of Grazeffe et al. [25], we obtain the MLE with only *X* and $X^{2}$ as covariates. We use the reflected Weibull link, because this model has lowest values of KS and MAE than those for Weibull link. To obtain the estimation of the reflected Weibull model we first estimate the values of $\theta_{1}$, $\theta_{2}$, $\theta_{3}$. To simplify, consider the three binary variables $Z_{1}$, $Z_{2}$ and $Z_{3}$, where $\theta_{k}=\mathrm{Pr}\left(Z_{k}=k\right)$, k=1,2,3. Then, using the results in Section 3.2, we construct Table 7 with the observed values of *Z*s, and estimate the models for *Z*s.

The parameter estimates for the three binary models are presented in Table 8, and we have $\hat{\theta_{1}}\left(x\right)=e^{-{\left(0.0234-1.6395x+0.6748x^{2}\right)}^{0.1742}}$, $\hat{\theta_{2}}\left(x\right)=e^{-{\left(1.0930-0.0368x+0.0030x^{2}\right)}^{2.3604}}$ and $\hat{\theta_{3}}\left(x\right)\;=\;e^{-{\left(1.2429-0.0866x+0.0047x^{2}\right)}^{1.7562}}$.

As described in Section 3.2, we have $\hat{p_{1}}\left(x\right)=\hat{\theta_{1}}\left(x\right)$, $\hat{p_{2}}\left(x\right)=$ $\left[1-\hat{\theta_{1}}\left(x\right)\right]\hat{\theta_{2}}\left(x\right)$, $\hat{p_{3}}\left(x\right)=\left[1-\hat{\theta_{1}}\left(x\right)\right]\left[1-\hat{\theta_{2}}\left(x\right)\right]\hat{\theta_{3}}\left(x\right)$, and $\hat{p_{4}}\left(x\right)=$ $\left[1-\hat{\theta_{1}}\left(x\right)\right]\left[1-\hat{\theta_{2}}\left(x\right)\right]\left[1-\hat{\theta_{3}}\left(x\right)\right]$, where $\hat{p_{k}}\left(x\right)$ is the estimated value of Pr[Y=k|x], k=1,…,4.

Table 9 presents the inferential statistics for model comparisons. All statistics indicate a preference for Weibull link model. The main difference for the Stukel model was because Weibull model has three parameters fewer than the Stukel model in this multinomial example.

**Figure 5:**
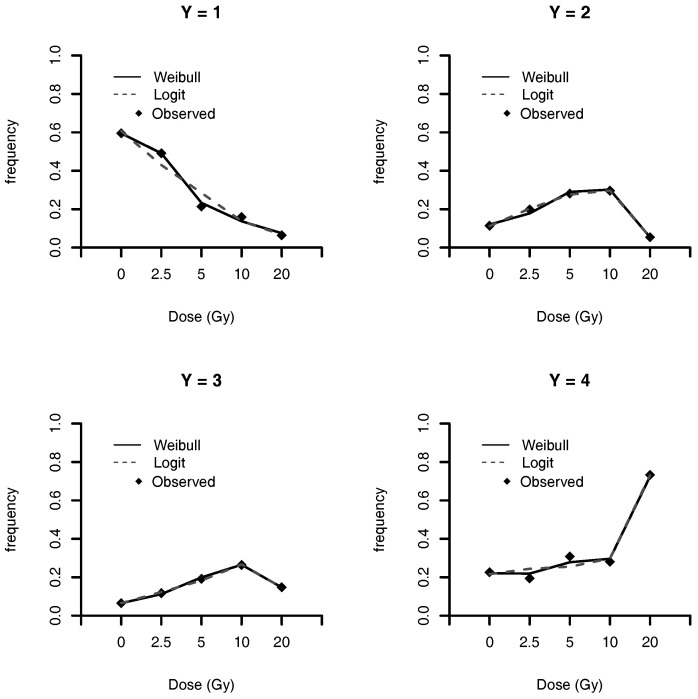
Estimated population frequencies.

The estimated frequencies, under Weibull, Stukel and logit models, of DNA mutation for each class is presented in Table 10, and illustrated in Figure 5. This figure shows that the Weibull link model has a better fit for categories Y=1 and 4, when compared with the logit link model. For categories Y=2 and 3, both models have comparable performances. Weibull and Stukel models have similar values of estimated frequencies.

## 5. Final Comments

In this paper, we have presented a Weibull model to estimate the problem of binary and multinomial regression analysis. The model is very flexible and capable to handle with many different types of data. The comparison with other skew-link model, in binomial data example (Section 4.1), shows that the performance of the Weibull link was good when compared to the others models. The model with worst measures was the Prentice model. All others had an equivalent result. We are convinced that our proposed model is a good option. A good feature of the model is that the logit, probit, complementary log–log, and log–log link functions are approximations of Weibull link. Then, the proposed model can accommodate even symmetric link function. For the flexibility of the Weibull link model, we are comfortable to suggest its use in practice.

Other aspect of the proposed Weibull model is that the associated numerical procedure of MLE is very simple to implement, particularly in comparison to other competing. For Bayesian estimates, we also suggest an Empirical Bayes approach to determine the prior. Under full Bayesian estimation, we compare the model with the skew-probit model [5] and AEP model [17], in Section 4.1. Again, all models had similar results, however the KS of Weibull model were the measures with the greatest differences among all models. The performance of our model was good, even under full Bayesian framework, in binomial data example (Section 4.1).

We also develop a partition scheme for the multinomial regression model simplifying the problem to K−1 binomial regression analysis. This is a general scheme that can be used for other link functions, which opens a vast options to estimate multinomial data. In Section 4.2, we analyze a multinomial data problem, where the Weibull model had the best measure values when compared with all other models. Our perspective is that the Weibull model is a good option for binary/multinomial regression, mainly due to its simplicity. We have analytic form for the link function, as well as for the gradient and Hessian matrix.

## Figures and Tables

**Table 1:** Link functions.

	Link Function	Parameteric Space
Weibull	$g^{-1}\left(\eta \right)=1-\exp\left(-\left(\eta^{\gamma }\right)\right)$	γ>0, η>0
reflected Weibull	$g^{-1}\left(\eta \right)=\exp\left(-\left(\eta^{\gamma }\right)\right)$	γ>0, η>0
AEP	if η≤0, take $\omega ={\left(-2\eta \Gamma \left(1+1/\theta_{1}\right)\right)}^{\theta_{1}}$ $g^{-1}\left(\eta \right)=\eta \exp\left(-\omega \right)/4+F_{G}\left(\omega ,1+1/\theta_{1},1\right)/2$	$\theta_{1}>0$ and $\theta_{2}>0$
	if η>0, take $\omega ={\left(2\eta \Gamma \left(1+1/\theta_{2}\right)\right)}^{\theta_{2}}$ $g^{-1}\left(\eta \right)=0.5+\eta \exp\left(-\omega \right)/4+F_{G}\left(\omega ,1+1/\theta_{2},1\right)/2$	
Aranda–Ordaz	$g^{-1}\left(\eta \right)=1-{\left(\alpha \exp\left(\eta \right)+1\right)}^{-1/\alpha }$	α>0
complementary log–log	$g^{-1}\left(\eta \right)=1-\exp\left(-\exp\left(\eta \right)\right)$	
log–log	$g^{-1}\left(\eta \right)=\exp\left(-\exp\left(\eta \right)\right)$	
logit	$g^{-1}\left(\eta \right)=\frac{\exp(\eta )}{1+\exp(\eta )}$	
Prentice	$g^{-1}\left(\eta \right)=F_{B}\left(\frac{1}{1+\exp\{-\eta \}},\lambda_{1},\lambda_{2}\right)$	$\lambda_{1}>0$ and $\lambda_{2}>0$
probit	$g^{-1}\left(\eta \right)=F_{N}\left(\eta \right)$	
skew-probit	$g^{-1}\left(\eta \right)=F_{SN}\left(\eta ,\delta \right)$	δ∈(−1,1)
Stukel	if η>0 and $\alpha_{1}>0$, $h\left(\eta \right)=\frac{\exp(\alpha_{1}\eta )-1}{\alpha_{1}}$	
	if η>0 and $\alpha_{1}=0$, h(η)=η	
	if η>0 and $\alpha_{1}<0$, $h\left(\eta \right)=\frac{-\log(1-\alpha_{1}\eta )}{\alpha_{1}}$	
	if η<0 and $\alpha_{2}>0$, $h\left(\eta \right)=\frac{-\exp(-\alpha_{2}\eta )-1}{\alpha_{2}}$	
	if η<0 and $\alpha_{2}=0$, h(η)=η	
	if η<0 and $\alpha_{2}<0$, $h\left(\eta \right)=\frac{\log(1+\alpha_{2}\eta )}{\alpha_{2}}$	
	$g^{-1}\left(\eta \right)=\frac{1}{1+\exp\{-h(\eta )\}}$	

AEP is the asymmetric exponential power link from Naranjo et al. [17]; Γ(·) is the mathematical gamma function; $F_{G}\left(\cdot ,a,b\right)$ is the distribution function of a random variable with distribution Gamma with shape *a* and scale *b*; $F_{B}\left(\cdot ,\lambda_{1},\lambda_{1}\right)$ is the distribution function of a random variable with distribution Beta$\left(\lambda_{1},\lambda_{2}\right)$; $F_{N}\left(\cdot \right)$ is the distribution function of a random variable with distribution normal, with mean zero and variance 1; and $F_{SN}\left(\cdot ,\delta \right)$ is the distribution function of a random variable with distribution skew-normal, with mean zero, variance 1 and asymmetric parameter δ [38].

**Table 2:** Relative potency of Rotenone, a Deguelin concentrate, and a Mixture of two.

Rotenone			Deguelin			Mixture		
log (Dose)	Dead	*n*	log (Dose)	Dead	*n*	log (Dose)	Dead	*n*
1.01	44	50	1.70	48	48	1.40	48	50
0.89	42	49	1.61	47	50	1.31	43	46
0.71	24	46	1.48	47	49	1.18	38	48
0.58	16	48	1.31	34	48	1.00	27	46
0.41	6	50	1.00	18	48	0.71	22	46
-	-	-	0.71	16	49	0.40	7	47

**Table 3:** Comparison of the link functions under MLE.

Model	log(L)	AIC	BIC	KS	MAE	B-S
Stukel	**−369.51**	751.01	779.26	0.1704	**0.0482**	**0.1474**
probit	−369.66	751.32	779.56	0.1583	0.0492	0.1475
comp. log–log	−370.32	**748.66**	**767.48**	0.1451	0.0551	0.1476
Weibull	−370.34	750.69	774.22	0.1440	0.0553	0.1477
logit	−372.57	753.14	771.97	**0.1292**	0.0656	0.1486
Aranda–Ordaz	−373.41	754.82	773.65	0.1351	0.0668	0.1487
Prentice	−374.90	759.80	783.33	0.3674	0.1396	0.1750

**Table 4:** MLE for the binomial example.

Parameter	Model [Estimate (SE)]		
	Weibull	Logit	Stukel
$\beta_{0}$	0.9735 (0.0110)	−3.9559 (0.3546)	−5.1973 (2.0524)
$\beta_{1}$	0.0266 (0.0111)	4.8273 (0.3394)	5.5892 (1.9677)
$\beta_{2}$	0.0053 (0.0024)	0.6910 (0.2308)	1.3233 (0.6397)
$\beta_{3}$	−0.0051 (0.0024)	−0.9125 (0.2449)	−1.0658 (0.4397)
-	γ= 114.5084 (47.9818)	-	$\alpha_{1}=$ 0.1732 (0.2871)
-	-	-	$\alpha_{2}=$−0.9663 (0.8492)

**Table 5:** Comparison of the link functions under Bayesian estimation.

	DIC	KS	MAE	B-S	Pr(D|M)
AEP	**749.01**	0.1826	**0.0564**	**0.1472**	744.66
Weibull	751.38	**0.1278**	0.0644	0.1484	748.42
skew-probit	751.92	0.1432	0.0662	0.1486	**749.13**

**Table 6:** Bayesian estimates for binomial example.

Parameter	Model [Posterior Mean (Standard Deviation)]		
	Weibull	Skew-Probit	AEP
$\beta_{0}$	0.2661 (0.2022)	−2.1900 (0.4006)	−4.9790 (1.5058)
$\beta_{1}$	0.7799 (0.2332)	2.6464 (0.2226)	5.2736 (1.4799)
$\beta_{2}$	0.1152 (0.0402)	0.3756 (0.1258)	1.2292 (0.4575)
$\beta_{3}$	−0.1474 (0.0576)	−0.4982 (0.1309)	−1.0145 (0.3830)
-	γ= 4.0285 (1.1992)	δ=−0.0434 (0.5506)	$\theta_{1}=$ 0.4491 (0.1152)
-	-	-	$\theta_{2}=$ 0.9057 (0.1904)

**Table 7:** Observed values of constructed variables $Z_{1}$, $Z_{2}$ and $Z_{3}$.

Dose (*X*)	$Z_{1}$		$Z_{2}$		$Z_{3}$	
	0	1	0	1	0	1
0	446	654	321	125	249	72
2.5	458	442	280	178	175	105
5	703	197	450	253	277	173
10	841	159	545	296	281	264
20	842	58	793	49	660	133

**Table 8:** MLE of Weibull model for the multinomial example.

Parameter	Model [Estimate (SE)]		
	$Y=1\;(Z_{1})$	$Y=2\;(Z_{2})$	$Y=3\;(Z_{3})$
γ	0.1742 (0.0209)	2.3604 (0.1905)	1.7562 (0.1415)
$\beta_{0}$	0.0234 (0.0123)	1.0930 (0.0258)	1.2429 (0.0369)
$\beta_{1}$	−1.6395 (0.8355)	−0.0368 (0.0067)	−0.0866 (0.0078)
$\beta_{2}$	0.6748 (0.3295)	0.0030 (0.0002)	0.0047 (0.0003)

**Table 9:** Comparison of the link functions for multinomial example.

	log(L)	AIC	BIC	KS	MAE	B-S
Weibull	**−5654.224**	**11332.45**	**11410.16**	**0.030**	**0.0095**	**0.6324**
Stukel	−5654.961	11339.92	11437.07	0.031	0.0100	0.6325
Prentice	−5667.156	11364.31	11461.46	0.066	0.0158	0.6341
Aranda–Ordaz	−5671.681	11361.36	11419.65	0.733	0.3116	1.1898
logit	−5672.196	11362.39	11420.68	0.075	0.0171	0.6348
comp. log–log	−5672.799	11363.60	11421.89	0.936	0.3129	1.2876
probit	−5673.003	11364.01	11422.29	0.079	0.0175	0.6348
log–log	−5676.075	11370.15	11428.44	0.693	0.2099	0.9490

**Table 10:** Relative frequencies of mutation (observed and model’s estimates).

Dose (Gy)	Model	Mutation Classes (*Y*)			
		1	2	3	4
0	Observed	0.595	0.114	0.065	0.226
	Weibull	0.595	0.120	0.065	0.220
	Logit	0.606	0.114	0.064	0.215
	Stukel	0.594	0.120	0.065	0.221
2.5	Observed	0.491	0.198	0.117	0.194
	Weibull	0.491	0.178	0.112	0.219
	Logit	0.430	0.201	0.123	0.246
	Stukel	0.490	0.178	0.113	0.218
5	Observed	0.214	0.281	0.192	0.308
	Weibull	0.233	0.289	0.200	0.278
	Logit	0.289	0.273	0.183	0.255
	Stukel	0.233	0.289	0.201	0.277
10	Observed	0.159	0.296	0.264	0.281
	Weibull	0.137	0.302	0.264	0.296
	Logit	0.136	0.298	0.268	0.298
	Stukel	0.135	0.304	0.263	0.298
20	Observed	0.064	0.054	0.148	0.733
	Weibull	0.075	0.054	0.147	0.725
	Logit	0.067	0.055	0.148	0.730
	Stukel	0.077	0.054	0.146	0.723

## Appendix A. Gradient and Hessian for Log-Likelihood of Weibull Model

Let $\xi_{i1}=\exp\left\{-\eta_{i}^{\gamma }\right\}$, $\xi_{i2}=\exp\left\{-2\eta_{i}^{\gamma }\right\}$, i=1,…,n.

### Appendix A.1. Gradient

The gradient vector is $g=(g_{1},g_{2},\ldots ,g_{r})$, where

$$\begin{array}{ccc} g_{1}=\frac{\partial l(\gamma ,\beta |D)}{\partial \gamma } & = & \sum_{i=1}^{n}-\left(1-y_{i}\right)\eta_{i}^{\gamma }\log\left(\eta_{i}\right)+\frac{y_{i}\xi_{i1}\eta_{i}^{\gamma }\log\left(\eta_{i}\right)}{1-\xi_{i1}}; \end{array}$$

$$\begin{array}{ccc} g_{2}=\frac{\partial l(\gamma ,\beta |D)}{\partial \beta_{0}} & = & \sum_{i=1}^{n}-\gamma \left(1-y_{i}\right)\eta_{i}^{\gamma -1}+\frac{\gamma y_{i}\xi_{i1}\eta_{i}^{\gamma -1}}{1-\xi_{i1}}; \end{array}$$

for j=1,…,r−2,

$$\begin{array}{ccc} g_{\left(j+2\right)}=\frac{\partial l(\gamma ,\beta |D)}{\partial \beta_{j}} & = & \sum_{i=1}^{n}-\gamma x_{ij}\left(1-y_{i}\right)\eta_{i}^{\gamma -1}+\frac{\gamma x_{ij}y_{i}\xi_{i1}\eta_{i}^{\gamma -1}}{1-\xi_{i1}}. \end{array}$$

### Appendix A.2. Hessian Matrix

The Hessian matrix H is

$$H=\left[\begin{array}{cccc} h_{11} & h_{12} & \cdots & h_{1r}\\ h_{21} & h_{22} & \cdots & h_{2r}\\ \vdots & \vdots & \ddots & \vdots \\ h_{r1} & h_{r2} & \cdots & h_{rr} \end{array}\right],$$

where, for j=1,…,r−2,

$$\begin{array}{ccc} h_{11}=\frac{\partial^{2}l\left(\gamma ,\beta |D\right)}{\partial \gamma \partial \gamma } & = & \sum_{i=1}^{n}-\left(1-y_{i}\right){\left[\log\left(\eta_{i}\right)\right]}^{2}\eta_{i}^{\gamma }\\ & & \;\;\;\;\;+\frac{\left\{\xi_{i1}y_{i}{\left[\log\left(\eta_{i}\right)\right]}^{2}\right\}\left[\eta_{i}^{\gamma }-\eta_{i}^{2\gamma }\right]}{1-\xi_{i1}}\\ & & \;\;\;\;\;-\frac{\xi_{i2}y_{i}{\left[\log\left(\eta_{i}\right)\right]}^{2}\eta_{i}^{2\gamma }}{{\left(1-\xi_{i1}\right)}^{2}}; \end{array}$$

$$\begin{array}{ccc} h_{21}=\frac{\partial^{2}l\left(\gamma ,\beta |D\right)}{\partial \gamma \partial \beta_{0}} & = & \frac{\partial^{2}l\left(\gamma ,\beta |D\right)}{\partial \beta_{0}\partial \gamma }=h_{12}; \end{array}$$

$$\begin{array}{ccc} h_{(j+2)1}=\frac{\partial^{2}l\left(\gamma ,\beta |D\right)}{\partial \gamma \partial \beta_{j}} & = & \frac{\partial^{2}l\left(\gamma ,\beta |D\right)}{\partial \beta_{j}\partial \gamma }=h_{1(j+2)}; \end{array}$$

$$\begin{array}{ccc} h_{12}=\frac{\partial^{2}l\left(\gamma ,\beta |D\right)}{\partial \beta_{0}\partial \gamma } & = & \sum_{i=1}^{n}-\left[1+\gamma \log\left(\eta_{i}\right)\right]\left(1-y_{i}\right)\eta_{i}^{\gamma -1}\\ & & \;\;\;\;\;+\frac{\left[1+\left(1-\eta_{i}^{\gamma }\right)\gamma \log\left(\eta_{i}\right)\right]\xi_{i1}y_{i}\eta_{i}^{\gamma -1}}{1-\xi_{i1}}\\ & & \;\;\;\;\;-\frac{\xi_{i2}\gamma y_{i}\log\left(\eta_{i}\right)\eta_{i}^{2\gamma -1}}{{\left(1-\xi_{i1}\right)}^{2}}; \end{array}$$

$$\begin{array}{ccc} h_{22}=\frac{\partial^{2}l\left(\gamma ,\beta |D\right)}{\partial \beta_{0}\partial \beta_{0}} & = & \sum_{i=1}^{n}-\left(\gamma -1\right)\gamma \left(1-y_{i}\right)\eta_{i}^{\gamma -2}\\ & & \;\;\;\;\;+\frac{\left[\left(\gamma -1\right)-\gamma \eta_{i}^{\gamma }\right]\xi_{i1}\gamma y_{i}\eta_{i}^{\gamma -2}}{1-\xi_{i1}}\\ & & \;\;\;\;\;-\frac{\xi_{i2}\gamma^{2}y_{i}\eta_{i}^{2\gamma -2}}{{\left(1-\xi_{i1}\right)}^{2}}; \end{array}$$

$$\begin{array}{ccc} h_{(j+2)2}=\frac{\partial^{2}l\left(\gamma ,\beta |D\right)}{\partial \beta_{0}\partial \beta_{j}} & = & \sum_{i=1}^{n}-\left(\gamma -1\right)\gamma x_{ij}\left(1-y_{i}\right)\eta_{i}^{\gamma -2}\\ & & \;\;\;\;\;+\frac{\left[\left(\gamma -1\right)-\gamma \eta_{i}^{\gamma }\right]\xi_{i1}\gamma x_{ij}y_{i}\eta_{i}^{\gamma -2}}{1-\xi_{i1}}\\ & & \;\;\;\;\;-\frac{\xi_{i2}\gamma^{2}x_{ij}y_{i}\eta_{i}^{2\gamma -2}}{{\left(1-\xi_{i1}\right)}^{2}}; \end{array}$$

$$\begin{array}{ccc} h_{1(j+2)}=\frac{\partial^{2}l\left(\gamma ,\beta |D\right)}{\partial \beta_{j}\partial \gamma } & = & \sum_{i=1}^{n}-\left[x_{ij}\left(1-y_{i}\right)\eta_{i}^{\gamma -1}\right]\left[1+\gamma \log\left(\eta_{i}\right)\right]\\ & & \;\;\;\;\;+\frac{\xi_{i1}x_{ij}y_{i}\eta_{i}^{\gamma -1}\left[1+\gamma \log\left(\eta_{i}\right)\left(1-\eta_{i}^{\gamma }\right)\right]}{1-\xi_{i1}}\\ & & \;\;\;\;\;-\frac{\xi_{i2}\gamma x_{ij}y_{i}\log\left(\eta_{i}\right)\eta_{i}^{2\gamma -1}}{{\left(1-\xi_{i1}\right)}^{2}}; \end{array}$$

$$\begin{array}{ccc} h_{2(j+2)}=\frac{\partial^{2}l\left(\gamma ,\beta |D\right)}{\partial \beta_{j}\partial \beta_{0}} & = & \frac{\partial^{2}l\left(\gamma ,\beta |D\right)}{\partial \beta_{0}\partial \beta_{j}}=h_{(j+2)2}; \end{array}$$

for k=1,…,r−2 and k≠j,

$$\begin{array}{ccc} h_{\left(k+2)(j+2\right)}=\frac{\partial^{2}l\left(\gamma ,\beta |D\right)}{\partial \beta_{j}\partial \beta_{k}} & = & \sum_{i=1}^{n}-\left(\gamma -1\right)\gamma x_{ij}x_{ik}\left(1-y_{i}\right)\eta_{i}^{\gamma -2}\\ & & \;\;\;\;\;+\frac{\left[\left(\gamma -1\right)-\gamma \eta_{i}^{\gamma }\right]\xi_{i1}\gamma x_{ij}x_{ik}y_{i}\eta_{i}^{\gamma -2}}{1-\xi_{i1}}\\ & & \;\;\;\;\;-\frac{\xi_{i2}\gamma^{2}x_{ij}x_{ik}y_{i}\eta_{i}^{2\gamma -2}}{{\left(1-\xi_{i1}\right)}^{2}}=h_{\left(j+2)(k+2\right)}. \end{array}$$
